# The Effects of Icariin on Enhancing Motor Recovery Through Attenuating Pro-inflammatory Factors and Oxidative Stress via Mitochondrial Apoptotic Pathway in the Mice Model of Spinal Cord Injury

**DOI:** 10.3389/fphys.2018.01617

**Published:** 2018-11-16

**Authors:** Haotian Li, Xinran Zhang, Xu Zhu, Xi Qi, Kaili Lin, Liming Cheng

**Affiliations:** ^1^Department of Spine Surgery, Tongji Hospital, Tongji University School of Medicine, Shanghai, China; ^2^Key Laboratory of Spine and Spinal Cord Injury Repair and Regeneration, Ministry of Education, Tongji University, Shanghai, China; ^3^School & Hospital of Stomatology, Shanghai Engineering Research Center of Tooth Restoration and Regeneration, Tongji University, Shanghai, China; ^4^Shanghai Key Laboratory of Stomatology, Shanghai Research Institute of Stomatology, Department of Oral and Craniomaxillofacial Surgery, Shanghai Ninth People’s Hospital, Shanghai Jiao Tong University School of Medicine, Shanghai, China

**Keywords:** spinal cord injury, icariin, mitochondria, apoptosis, oxidative stress, inflammation

## Abstract

Spinal cord injury (SCI) is a severe medical problem leading to crucial life change. Icariin (ICA) is a natural flavonoid compound extracted from the Chinese herb Epimedium brevicornum which has neuroprotective effects. But little is known about the relationship between ICA and SCI. We hypothesized ICA may enhance motor recovery through attenuating inflammation, oxidative stress and mitochondrial dysfunction. Mice were randomly assigned to sham, SCI, ICA 20 μmol/kg (low dose) and ICA 50 μmol/kg (high dose) groups. And Behavioral, biochemical, molecular biological, immunofluorescent and histological assays were performed. First, ICA enhanced motor recovery greatly at 14, 28, and 42 days and protected spinal cord tissues especially in the high dose group. Meanwhile, ICA decreased the production of interleukin-1 beta, tumor necrosis factor-alpha and inducible nitric oxide synthase at 24 h and 3 days after SCI. The level of mitochondrial reduced glutathione, superoxide dismutase, adenosine triphosphate (ATP), Na^+^-K^+^-ATPase, mitochondrial membrane potential, state III respiration rate and the respiratory control ratio were also significantly increased, while malondialdehyde level and Ca^2+^ concentration were decreased by ICA. Furthermore, ICA decreased the expression of mitochondrial apoptotic proteins at 3 days after SCI. More importantly, transferase UTP nick end labeling (TUNEL) and Nissl staining implied that ICA at a high dose inhibited the neuronal apoptosis after SCI. Our research indicated that early and continuous treatment of ICA at a high dose significantly enhanced motor recovery after SCI through inhibiting pro-inflammatory factors, oxidative stress and neuronal apoptosis via mitochondrial apoptotic pathway.

## Introduction

Spinal cord injury (SCI) is a severe neurological disease and challenging issue both for scientists and clinicians. There were nearly 17,000 new cases of SCI in the United States by the end of 2016 based on the study from the National Spinal Cord Injury Center website. And the annual incidence is approximately 40 patients per million (Rabchevsky et al., 2012; Anonymous, 2016). The majority of patients with SCI are males younger than age 30 years which typically leads to progressive complications both physical and psychological. SCI and its consequences place a tremendous burden on individuals, family and society (Chen et al., 2015). The main reason leads to disability and poor functional recovery is a complex pathophysiological process of secondary damage following the irreversible primary injury. Although many researchers have been struggling to illustrate the underlying mechanisms of SCI, no effective medical therapy currently exists.

Over the past few years, mounting evidences suggest that mitochondrial function, oxidative stress and inflammatory response contribute significantly to SCI-induced secondary damage. Based on this evidence, we speculated that therapies targeting a specific signaling pathway such as oxidative stress may not provide the best efficacy for treating SCI. Therefore, we put forward the conception of “systemic organelles therapies” which maybe a better strategy for SCI via modulating mitochondria.

Mitochondria are the “energy factories” and involve in oxidative stress, apoptosis, endoplasmic reticulum (ER) stress and so on (Beigi Harchegani et al., 2018). Accumulating studies showed that mitochondrial dysfunction led to an imbalance between oxidants and antioxidants in SCI (Li H. et al., 2015). Furthermore, the inhibition of ATP production, the mitochondrial Ca^2+^ overload, the decrease of mitochondrial respiratory control ratio (RCR) and the depolarization of the mitochondrial membrane potential (MMP) often result in severe inflammatory response and neuronal apoptosis.

Inflammation regulates the immune response and participates in various physiological processes. However, the excessive inflammatory response after SCI induces a significant increase in leucocytes and cytokines including interleukin-1 beta (IL-1β) and tumor necrosis factor-alpha (TNF-α), resulting in the infiltration of leucocytes to the injured location and further tissue damage. Although the crosstalk between inflammation and mitochondria still remains unclear, the increase production of inflammatory cytokines following SCI may be responsible for neurological disorders in the secondary damage.

Icariin (ICA) is a natural flavonoid compound isolated from famous Chinese herb Epimedium brevicornum, also known as Yin Yanghuo. Extracts from Epimedium are traditional medical therapies for rheumatic disease and also used as aphrodisiacs in China (Chen et al., 2016). As the major active component of Epimedium, ICA has been shown broad physiological functions including anti-oxidant, anti-inflammatory, immunoregulation, etc. Recent studies indicated that ICA had a protective role in various neurological models such as cerebral ischemia–reperfusion injury (Li et al., 2005), brain injury (Zhu et al., 2010), neuronal apoptosis (Liu et al., 2011), Alzheimer’s disease (Urano and Tohda, 2010) and neuronal regeneration (Li et al., 2010; Yang and Chen, 2013), etc. Various mechanisms are correlated with the neuroprotective effects of ICA. Li F. et al. (2015) observed that ICA inhibited neuronal apoptosis induced by ER stress in the study of neuronal degenerative disease. Meanwhile, the protective effect of ICA on ischemic stroke maybe related to the inhibition of inflammatory responses (Xiong et al., 2016). More importantly, the previous study has shown that ICA could inhibit the apoptosis of cardiomyocytes through inhibiting the mitochondrial apoptotic pathway (Qian et al., 2017). Furthermore, it has been shown that ICA may ameliorate blood viscosity and memory deficit by inhibiting oxidative stress in the model of brain ischemic (Zheng et al., 2010). Moreover, Wang et al illustrated that ICA reduced dopaminergic neuronal loss via inhibiting inflammation (Wang et al., 2017a). However, no studies have focused on the effects and mechanisms of ICA in the mice model of SCI so far. Hence, we investigated that whether ICA had a “systemic organelles therapeutic” effect through protecting mitochondria function, anti-oxidation and anti-inflammation in this study.

## Materials and Methods

### Animals

Adult male C57BL mice (8-week old) were purchased from the Experimental Animal Center of Tongji University (Shanghai, China). The use of mice in this study was approved by the Ethical Committee of Tongji Hospital. And all experimental procedures were conducted in accordance with the Guide for the Care and Use of Laboratory Animals of Tongji University. ICA was purchased from Shanghai Aladdin Biochemical Technology Co., Ltd., (purity 98.0%; molecular weight 676.66; Shanghai, China). The animals were divided into four groups with randomization method (randomization table assay): the sham group, SCI group, ICA 20 μmol/kg group, and ICA 50 μmol/kg group. The sham group was only performed a laminectomy without SCI, while the others were established the model of SCI. The different doses of ICA at 20 or 50 μmol/kg were given to the mice in the ICA groups by the oral route once daily post injury. Meanwhile, a vehicle solution (0.1% DMSO in 0.9% normal saline) was given orally once daily in the sham group and SCI group.

### Induction of SCI in Mice

The model of SCI was induced under sterile conditions. First, the mice were anesthetized using pentobarbital sodium (40 mg/kg) by intraperitoneal injection (i.p.). Briefly, a laminectomy was conducted at T9–10 without injuring the dura. SCI was induced by Infinite Horizon (IH) Spinal Cord Impactor (Precision Systems and Instrumentation, LLC. Springfield, NJ, United States) as described previously (Luchetti et al., 2010). The force was 60 k dynes, and the dwell time was 0 s. All mice received 0.9% normal saline (30 ml/kg, i.p.) to prevent dehydration after SCI. Bladders were manually expressed twice a day until the mice could urinate normally.

### Behavioral Tests

The Basso Mouse Scale (BMS) behavioral test in an open-field was performed at 0, 1, 3, 7, 14, 28, and 42 days after SCI according to the previous study (Basso et al., 2006). All animals were put into the open-field environment for 3 days (20 min/day) before the experiment. The testing environment was 50 cm wide × 50 cm long × 30 cm high in the scale. Each mouse was investigated by the researchers who are blinded to the groups and independent.

### The Water Content of Spinal Cord Tissue

The perilesional spinal cord tissues (epicenter ± 0.5 cm) were harvested, weighed, dried at 80°C for 2 days and weighed again. (wet weight-dry weight)/wet weight × 100% was calculated to detect the edema at 24 h and 3 days after SCI.

### Enzyme-Linked Immunosorbent Assay (ELISA)

At 24 h and 3 days after injury, mice were sacrificed and then perfused via the aorta with 0.9% normal saline. The perilesional tissues of spinal cord were dissociated and homogenized with lysis buffer containing 2 mmol/L of phenylmethyl sulfonyl fluoride (PMSF, Beyotime, Jiangsu, China). After centrifugation for 15 min at 2000 ×*g* and 4°C, the level of TNF-α and IL-1β were detected by commercial ELISA Kit (Beyotime; Jiangsu, China). Finally, the absorbance at 450 nm was analyzed by fluorescence spectrophotometer (Shanghai Analytical Instrument Factory, 970CRT, China), and the concentrations were calculated.

### Preparation of Mitochondria and Cytoplasm of Spinal Cord Tissues

Mitochondria and cytoplasm of spinal cord tissues were isolated according to cytosol extraction and mitochondria isolation kit (Applygen Technologies Inc., Beijing, China). At 24 h or 3 days after injury, the tissues (epicenter ± 0.5 cm) were rinsed with phosphate-buffered saline (PBS) and cut into pieces in a homogenizer. The homogenate was centrifuged for 5 min at 800 ×*g* and 4°C. The supernatant was removed into another centrifuge tube and centrifuged again as previously. Then the supernatant was centrifuged for 10 min at 12,000 ×*g* and 4°C for the third time. Finally, the mitochondria deposits were present at the bottom of the tube and the cytoplasm was in the supernatant.

### Measurement of Mitochondrial MDA, SOD and GSH

The biochemical kits (Jiancheng Institute of Biology, Nanjing, China) were used to measure and normalize the level of MDA, SOD and reduced GSH according to the protocol of the kit restrictedly.

### Measurement of MMP by JC-1

MMP was detected by 5,5’,6,6’-tetrachloro-1,1’,3,3’-tetraethylbenzimidazolcarbocyanine (JC-1) Mitochondrial Membrane Potential Assay Kit (Abcam, Cambridge, MA, United States), fluorescence microscopy and fluorescence spectrophotometer. FCCP was used as the control during the measurement. The depolarization of MMP is correlated with an increase in green signals while a decrease in the red signals. The fluorescence was assessed by green (λ_exc= 488nm,λ_em_ = 530 nm) and red (λ_exc= 525nm,λ_em_ = 590 nm) wavelengths. Then the ratio between red and green fluorescence values was calculated.

### Measurement of Mitochondrial Energy, Ca^2+^ Concentration and Respiration

ATP production, Na^+^-K^+^-ATPase and Ca^2+^ concentration were detected according to the protocol of the kit restrictedly (Jiancheng Institute of Biology, Nanjing, China) at 3 days post injury. Mitochondrial ATP was expressed as μmol/g protein, Na^+^-K^+^-ATPase was expressed as U/mg protein, and Ca^2+^ concentration was expressed as mmol/ g protein. Mitochondrial respiration was detected in a controlled chamber by a miniature Clark-type electrode (Hansatech Instruments, Norfolk, England) and the results were expressed as nmols oxygen/min/mg protein as described previously (Patel et al., 2009). Furthermore, the RCR was calculated as the ratio of state III vs. state IV slopes.

### Western Blot

The tissues (epicenter ± 0.5 cm) were homogenized and extracted using RIPA buffer (Beyotime, Jiangsu, China) with PMSF at 3 days after SCI. After measured by the bicinchoninic acid (BCA) assay, the protein (30 μg/lane) was firstly separated through 10 or 15% sodium dodecyl sulfate polyacrylamide gel electrophoresis (SDS-PAGE), and then transferred to 0.2 or 0.45 μm polyvinylidene fluoride (PVDF) membranes (Millipore, Bedford, MA, United States). After blocking for 1 h at room temperature with 5% skimmed milk, the membranes were incubated with the following primary antibodies against Cyt C (Abcam, United States), Bcl-2 (CST, United States), Bax (CST, United States), caspase-3 (CST, United States), cleaved caspase-3 (CST, United States), caspase-9 (CST, United States), cleaved caspase-9 (CST, United States), PARP (CST, United States), cleaved PARP (CST, United States) and β-actin (CST, United States) overnight at 4°C. After washing with tris buffered saline tween (TBST), the membranes were incubated with the goat anti-rabbit IgG-HRP or goat anti-mouse IgG-HRP secondary antibodies (Abcam, United States) for 2 h at room temperature. The membranes illuminated with enhanced chemiluminescence substrate (Millipore, Billerica, MA, United States) were photographed with Image Quant LAS 4000 (GE, United States) and analyzed by Image J software (NIH, Bethesda, MD, United States).

### Tissues Preparation

The mice were firstly transcardially perfused under anesthesia with 20 ml 0.9% normal saline, and then 20 ml 4% paraformaldehyde (PFA) (in 0.1 M PBS, pH 7.4) at 3 days after injury. Spinal cord tissues (epicenter ± 0.5 cm) were taken and post-fixed for 24 h in 4% PFA overnight. Tissues were dehydrated sequentially with 20 and 30% sucrose solution at 4°C. Then tissues were embedded with Tissue-Tek O.C.T. compound (Sakura Finetechnical, Tokyo, Japan) and sliced with freezing microtome (Leica, Germany) at -20°C. Six sequential 5-μm thickness transverse sections at 200-μm intervals of each mouse were collected.

### Immunofluorescence and TUNEL Staining

Slices were permeabilized and blocked, respectively, with 0.1% Triton X-100 or 5% normal goat serum. The following primary antibodies against NeuN (Abcam, United States) caspase-3 (CST, United States) and inducible nitric oxide synthase (iNOS) (Millipore, Germany) were incubated overnight at 4°C. After washing with PBS for 3 times, the sections were probed with mixed secondary antibody Alexa Fluor^®^ 594 and 488 goat anti-rabbit IgG (H + L) (Life Technologies Corporation, United States) for 1 h at room temperature in the dark. Afterward, TUNEL staining was performed according to the instructions (Roche, United States). The samples were then counterstained with 4’, 6-diamidino-2-phenylindole (DAPI, Sigma Aldrich, United States) for 3 min. During the procedures, omission of the relevant primary antibody was performed for negative controls. All sections were observed using the confocal laser scanning microscope (CLSM 800, Zeiss, Germany). The number of positive neurons was counted in six randomly chosen fields from one section. Six mice were examined per group. The percentage of apoptotic neurons was calculated.

### HE Staining and Nissl Staining

For Nissl staining, 0.1% crystal violet (Sigma, United States) was used firstly, followed by differentiated by 95 and 100% alcohol. Then the slices were rinsed by xylene. For HE staining, the slices were stained according to the manufacturer’s protocol of HE staining kit (Beyotime, Jiangsu, China). After mounting, the sections were observed by Olympus light microscope (Olympus, Shanghai, China). The histopathologic changes and the numbers of survival neurons were observed and counted.

### Statistical Analysis

The data were expressed as mean ± standard error. Student’s *t*-test was used to compare only two groups. Multiple comparisons were evaluated by SNK test using one-way ANOVA (SPSS 16.0, United States). Values of *p* < 0.05 were considered statistically significant.

## Results

### ICA Enhanced Motor Recovery After SCI

**FIGURE 1:**
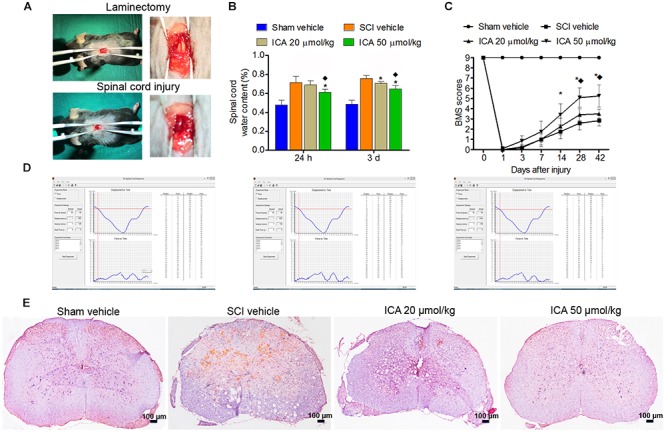
ICA protected spinal cord tissues and enhanced motor recovery after SCI. **(A)** The laminectomy and SCI. **(B)** The water content in SCI group was markedly increased. However, the water content in ICA 50 μmol/kg group at 1 day and 3 days was decreased. **(C)** BMS scores were notably decreased after SCI. BMS scores of the ICA 20 μmol/kg and 50 μmol/kg group increased, respectively, at 28, 42 days or 14, 28 and 42 days. Moreover, BMS scores of ICA 50 μmol/kg group increased significantly at 28 days and 42 days after SCI. **(D)** The information of mice model of SCI. **(E)** HE staining. Compared with the SCI group, ICA markedly relieved the tissue injury (Scale bars = 100 μm). *N* = 6. ^∗^*P*< 0.01 compared to SCI group; ^
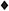
^*P*< 0.01 compared to 20 μmol/kg group.

As showed in Figure 1A, the dura and spinal cord tissue were intact and normal in the sham group. However, hemorrhage and edema were found in the SCI group. The displacement versus time and force versus time were all recorded by computer which ensured the repeatability and consistency of the model of SCI (Figure 1D).

Compared with the sham group, the BMS scores of SCI and ICA groups significantly decreased, and there was no statistical difference between these groups over 1-week period after SCI, which indicated SCI induced severe motor dysfunction (Figure 1C). However, the treatment of ICA at a dose of 20 μmol/kg increased BMS scores at 28 and 42 days after injury compared with SCI group. With respect to 50 μmol/kg group, BMS scores increased significantly at 14, 28, and 42 days post surgery. Furthermore, the BMS score of ICA 50 μmol/kg group was increased remarkably compared with that of 20 μmol/kg group at 28 days and 42 days after operation. In contrast to low dose of ICA, the results suggested that higher doses of ICA may better enhance motor recovery after SCI.

### ICA Protected the Spinal Cord Tissues and Reduced Edema After SCI

In the sham group, the samples exhibited clear boundary and integrated morphology. However, there were more eosinophilic neurons, hemorrhage, and larger infarction in the SCI group. Compared with SCI group, tissue morphology of ICA treatment group especially the 50 μmol/kg group was relevantly maintained with clear boundaries and normal morphology (Figure 1E). Furthermore, the reactive glial cells and the area of infarction decreased in the ICA 50 μmol/kg group compared with 20 μmol/kg group (Scale bars = 100 μm).

Furthermore, the water content in the SCI group was significantly elevated as shown in Figure 1B. And there was no statistical difference between ICA 20 μmol/kg group and SCI group at 24 h. However, the edema was significantly reduced after 1 and 3 days of 50 μmol/kg ICA treatment and 3 days of 20 μmol/kg ICA treatment. Moreover, the mice treated with 50 μmol/kg ICA exhibited significant reduction in edema at all time points compared with those in the ICA 20 μmol/kg group.

### ICA Attenuated Inflammation and Oxidative Stress

**FIGURE 2:**
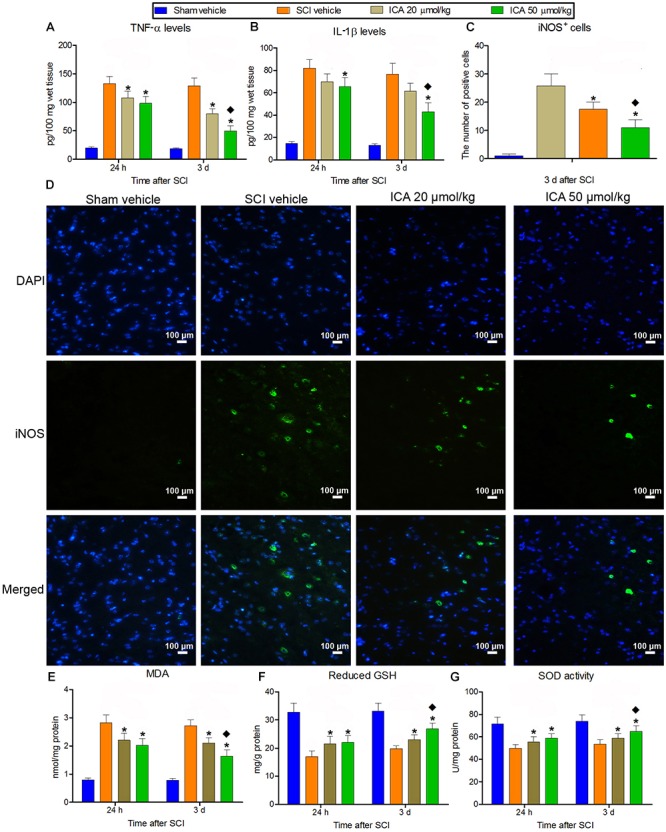
The measurement of inflammation and oxidative stress. **(A–D)** IL-1β and TNF-α production and the number of iNOS^+^ cells markedly increased after SCI at 24 h and 3 days. But the inflammation was relieved by ICA. And the inflammatory response was significantly inhibited in 50 μmol/kg group. **(E–G)** The level of reduced GSH and SOD decreased, while the content of MDA increased significantly at 24 and 3 d post injury. The treatment of ICA attenuated oxidative stress at all time points. Moreover, the oxidative stress was better relieved in the 50 μmol/kg group than the 20 μmol/kg group. *N* = 6. ^∗^*P*< 0.01 compared to SCI group; ^
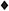
^*P*< 0.01 compared to 20 μmol/kg group.

A significant increase in IL-1β, TNF-α and iNOS positive cells production was found in SCI group (Figures 2A–D). But the treatment of ICA decreased the concentration of IL-1β and TNF-α and the numbers of iNOS^+^ cells at 24 h and 3 days compared with SCI group. Furthermore, the inflammation decreased significantly in the 50 μmol/kg group compared with the 20 μmol/kg group.

Besides, the level of reduced GSH and SOD decreased significantly, while the content of MDA increased significantly at 24 h and 3 days post injury (Figures 2E–G). But ICA reversed this trend, and there was no statistical difference between ICA 20 μmol/kg group and 50 μmol/kg group at 24 h. However, the mice in the 50 μmol/kg ICA group exhibited a significant reduction in MDA but increase in reduced GSH and SOD levels compared with the ones in the 20 μmol/kg group at 3 days post injury.

### ICA Preserved Mitochondrial Function and Inhibited Mitochondrial Ca^2+^ Overload After SCI

**FIGURE 3:**
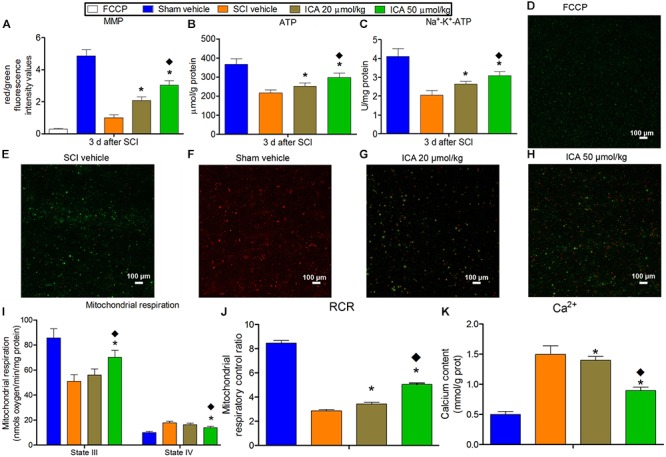
The evaluation of mitochondrial function. **(A–C** and **I–K)** In SCI group, the MMP, ATP production, Na^+^-K^+^-ATPase activity, state III respiration rate and RCR decreased significantly. And the concentration of Ca^2+^ and state IV respiration rate in mitochondria increased significantly after SCI. But the ICA treatment reversed these trends. Moreover, the mitochondrial function was significantly promoted in ICA 50 μmol/kg group. **(D–H)** JC-1 staining of MMP. *N* = 6. ^∗^*P*< 0.01 compared to SCI group; ^
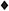
^*P*< 0.01 compared to 20 μmol/kg group.

The red fluorescence was markedly reduced in the FCCP control group and SCI group which indicated the depolarization of MMP after SCI (Figure 3). And ATP production, Na^+^-K^+^-ATPase activity, state III respiration rate and RCR also decreased significantly except for sham group. Besides, the concentration of Ca^2+^ and state IV respiration rate in mitochondria increased significantly after SCI which suggested the Ca^2+^ overload. However, MMP, ATP content, Na^+^-K^+^-ATPase activity, state III respiration rate and RCR in ICA 50 μmol/kg group significantly increased compared with that in ICA 20 μmol/kg group at 3 days post injury. While, the concentration of Ca^2+^ and state IV respiration rate in ICA 50 μmol/kg group were significantly decreased compared with that in ICA 20 μmol/kg group. Taken together, it suggested that the high dose treatment of ICA may have the better restoration of mitochondrial function after SCI.

### ICA Inhibited the Mitochondrial Apoptotic Pathway

**FIGURE 4:**
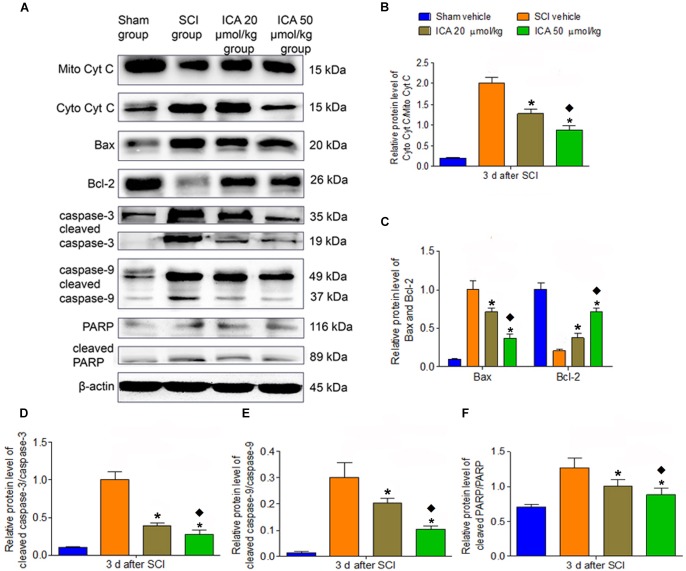
Western blot assay. **(A)** The expression of mitochondrial apoptotic proteins. **(B–F)** The relative level of cytoplasm Cyt C/mitochondria, Bax/Bcl-2, cleaved caspase-3/caspase-3, cleaved caspase-9/caspase-9 and cleaved PARP/PARP all significantly increased in the SCI group. However, the treatment of ICA decreased the relative level. The level of proteins was decreased significantly by ICA 50 μmol/kg. *N* = 6. ^∗^*P*< 0.01 compared to SCI group; ^
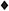
^*P*< 0.01 compared to 20 μmol/kg group.

As shown in Figure 4, the relative level of cytoplasm Cyt C/mitochondria Cyt C, Bax/Bcl-2 and caspase family proteins all significantly increased in the SCI group which indicated that the mitochondrial apoptotic pathway was active at 3 days post SCI. However, the treatment of ICA reversed this trend. Moreover, compared with ICA at the dose of 20 μmol/kg, the level of mitochondrial apoptotic proteins was decreased significantly by ICA at the dose of 50 μmol/kg. Consistent with the results above, we focused on the dose of 50 μmol/kg of ICA in the following study.

### ICA Decreased the Number of Caspase-3 Positive Neurons After SCI

**FIGURE 5:**
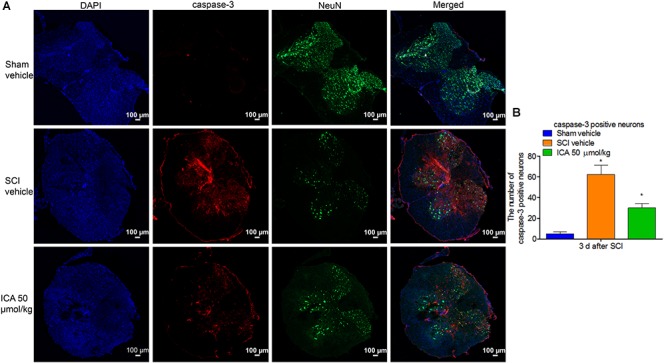
Immunofluorescence staining of caspase-3/NeuN/DAPI. **(A)** Immunofluorescence staining (Scale bars = 100 μm). **(B)** The number of caspase-3^+^ neurons was dramatically increased after SCI. However, 50 μmol/kg ICA significantly decreased caspase-3 positive neurons. *N* = 6. ^∗^*P*< 0.01 compared to SCI group.

As shown in Figure 5A, the caspase-3 showed red punctate caspase-3 dots, and NeuN showed green fluorescence. Therefore, caspase-3/NeuN/DAPI positive neurons displayed red fluorescence with blue nucleus and the green signals. As shown in Figure 5B, the number of caspase-3^+^ neurons was significantly increased after SCI. However, the expression was notably inhibited by ICA administration.

### ICA Inhibited the Neuronal Apoptosis After SCI

**FIGURE 6:**
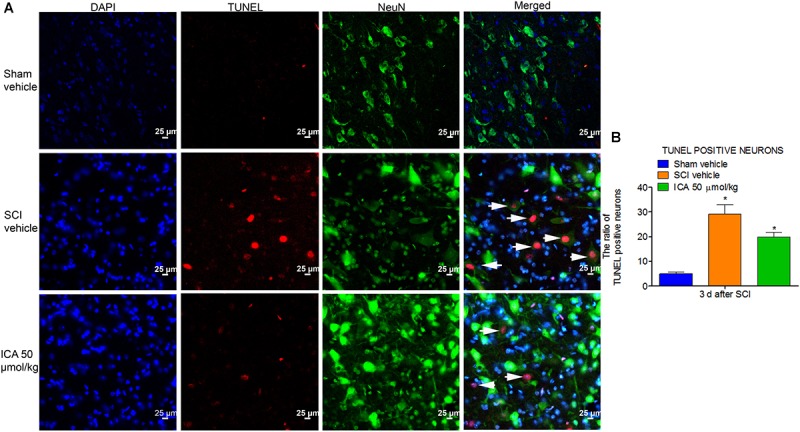
Immunofluorescence staining of TUNEL/NeuN/DAPI. **(A)** Immunofluorescence staining of TUNEL positive neurons. The white arrow indicted the apoptotic neurons (Scale bars = 25 μm). **(B)** The ratio of apoptotic neurons increased remarkably after SCI. However, high dose of ICA dramatically decreased the ratio of TUNEL positive neurons. *N* = 6. ^∗^*P*< 0.01 compared to SCI group.

As shown in Figure 6, the TUNEL/NeuN/DAPI positive neurons displayed red fluorescence with blue nucleus and the green signals. The figures showed that neuronal apoptosis was dramatically induced by SCI at 3 days post injury. However, ICA administration remarkably decreased the neuronal apoptosis ratio.

### ICA Increased the Number of Survived Neurons After SCI

**FIGURE 7:**
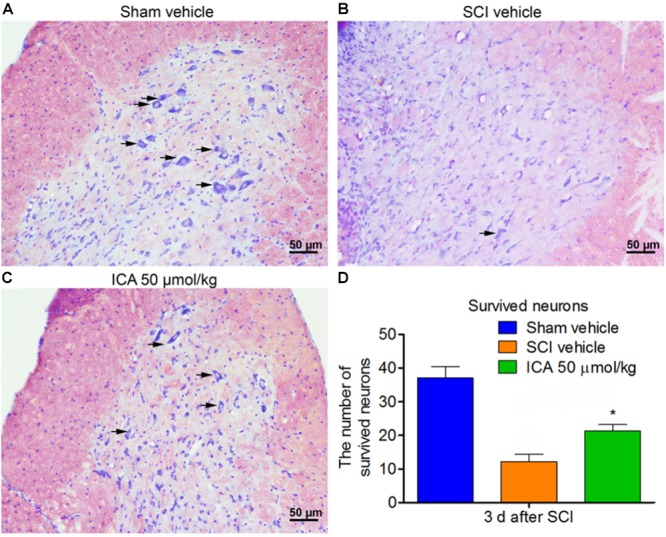
Nissl staining. **(A–C)** The survived neurons were detected by Nissl staining in different groups. ICA significantly reduced the area of cavity and infraction. The black arrow indicated the survived neurons (Scale bars = 50 μm). **(D)** The number of survived neurons in ICA group significantly increased. *N* = 6. ^∗^*P*< 0.01 compared to SCI group.

The neurons exhibited normal and integrative morphology in sham group (Figure 7A). However, in the SCI group, the slices exhibited connective tissue, abnormal morphologies and large cavity. The Nissl bodies and boundaries between gray and white matters became obscure, and the number of survived neurons was also remarkably decreased. However, ICA promoted neuronal survival in the ICA 50 μmol/kg group. The number of neurons with normal morphology and Nissl bodies increased in the ICA group, and the area of cavity and infraction was also smaller near the epicenter of the injury compared with the SCI group (Figures 7B–D).

### The Schematic

**FIGURE 8:**
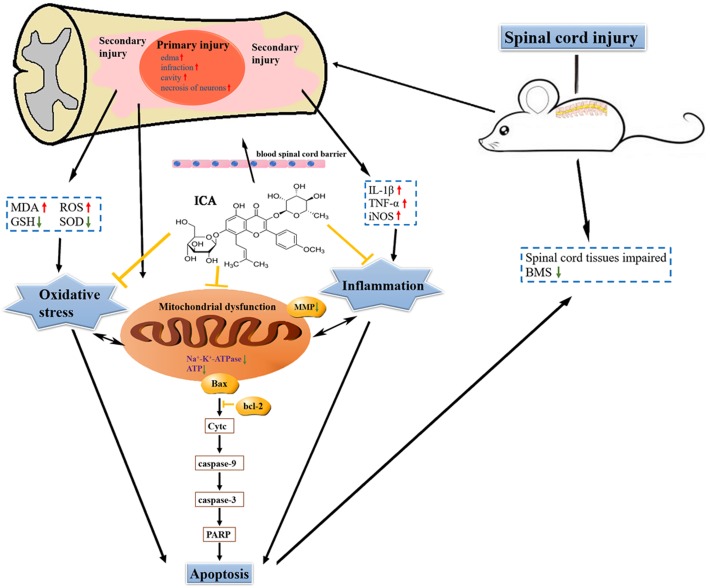
The schematic. ICA significantly inhibits oxidative stress, inflammation and neuronal apoptosis by inhibiting the mitochondrial apoptotic pathway after SCI.

The schematic shows that attenuating pro-inflammatory factors and oxidative stress via the inhibition of mitochondrial apoptotic pathway by ICA may be the underlying mechanisms of enhancing motor recovery in the mice model of SCI (Figure 8).

## Discussion

Icariin has a neuroprotective effect in both acute and chronic central nervous system disease (Fu et al., 2018). In our research, it demonstrated that ICA especially at a high dose promoted motor recovery after SCI. Xiong et al. (2016) confirmed that the pretreatment of ICA at a low dose of 10 mg/kg and a high dose of 30 mg/kg attenuated acute cerebral ischemia–reperfusion injury, while ICA at the high dose had the better effects. The results of Zhu et al. (2010) also indicated that ICA administration at higher doses could remarkably attenuate acute middle cerebral artery occlusion. What the most important was that ICA at a high dose of 50 μmol/kg improved motor recovery after SCI (Tohda and Nagata, 2012), and the similar results were also supported by other studies (Li et al., 2005; Guo et al., 2010). Therefore, we may come to the conclusion that ICA at a high dose may be a better therapeutic strategy according to our and other scholars’ researches.

To recapitulate the molecular mechanisms of secondary damage is a critical issue in the study of SCI (Sandrow-Feinberg and Houle, 2015; Yan et al., 2018). Autophagy (Li et al., 2016) and neuronal apoptosis via AMPK pathway (Wang et al., 2017b) have been studied by us, but few studies reported the therapies targeting on mitochondria in SCI. SCI-induced mitochondrial dysfunction leads to ROS accumulation and the increase of lipid peroxides (Bermudez et al., 2016). Because numerous lipids constitute spinal cord tissue, it is easily injured by lipid peroxidation and ROS. MDA is a stable metabolite of lipid peroxidation cascade. SOD and GSH are all important endogenous antioxidants (Genovese et al., 2007). Therefore, MDA, GSH and SOD are widely used as reliable indexes for determining the extent of peroxidation after SCI. As shown in Figure 2, the treatment of ICA increased GSH and SOD, but decreased the level of MDA at 24 h and 3 days after SCI. This is consistent with our previous research on the time-dependent mitochondrial function and morphology in SCI (Jia et al., 2016). What important is that, although there is no statistical difference between ICA 20 μmol/kg group and 50 μmol/kg group at 24 h after SCI, the mice treated with 50 μmol/kg ICA exhibited a significant reduction in MDA but increase in GSH and SOD at 3 days post injury. This implied that the anti-oxidation activity of ICA at 3 days was much stronger than that at 24 h. It suggested that not only the early intervention is necessary, but the continuous treatment of ICA is also important in the treatment of SCI.

Furthermore, the mitochondrial function was assessed to study the effect of ICA. Mitochondrial membrane consists of outer membrane containing voltage-dependent anion channels and inner membrane regulating the electron transport chain (ETC) to maintain the pivotal MMP for biological oxidation and ATP production (Gollihue et al., 2018; Kvirkvelia et al., 2018). In the early stage of SCI, the mitochondrial permeability transition pore (mPTP) is opened by accumulation of glutamate and intracellular Ca^2+^ through the anion channels (Savino et al., 2013). Consistent with our previous study, the opening of the mPTP after SCI will depolarize the MMP, induce Ca^2+^ overload, decrease RCR, release Cyt C and activate caspase family members (Li H. et al., 2015). However, the present results showed that ICA significantly increased MMP, RCR and ATP content, but inhibited Ca^2+^ overload compared with SCI group, suggesting that ICA preserved the mitochondrial function after SCI.

In addition to oxidative stress, excessive inflammatory cytokines including IL-1β and TNF-α contribute to the pathophysiology and neuronal apoptosis in SCI (Ferguson et al., 2008). TNF-α increases significantly in the stage of SCI and is a potent activator of neutrophils. IL-1β is a pro-inflammatory cytokine and has been indicated in excessive inflammation (Sahin et al., 2011). Therefore, our data supported strongly that IL-1β and TNF-α were significantly activated after 24 h and 3 days post injury. However, ICA significantly reversed this trend at a time-dependent manner. Hence, our results indicate that anti-inflammation is a potential underlying mechanism of neuroprotective effect of ICA.

Previous studies have indicated that neuronal loss and motor function impairment after SCI are significantly related to apoptosis mediated by mitochondrial pathway (Wu et al., 2007). Bcl-2 is a pivotal anti-apoptotic gene in the outer mitochondrial membrane which could inhibit Cyt C release and apoptosis (Lee et al., 2014). Bax is highly homologous with Bcl-2 but functions oppositely. After releasing from mitochondria to cytosolic, Cyt C activates the executioner caspase-3 which can lead to DNA fragmentation and other cascades of apoptotic process. And the cleaved caspase-3 protease induces the execution of apoptosis via PARP (Scott et al., 2004). As showed in Figure 4, the expressions of the apoptotic proteins were all inhibited by ICA at 3 days post injury, which is consistent with the results of anti-inflammation and anti-oxidation. Furthermore, the triple labeling immunofluorescence staining was used to analyze the neuronal apoptosis (Figures 5, 6). This method is more specific than before because there are various types of cells except for neurons in spinal cord. Notably, the number of caspase-3 positive neurons was decreased by ICA compared with SCI group. And the same trend shown in the TUNEL staining also suggested that ICA decreased the number of apoptotic neurons. In brief, the results indicated that ICA significantly inhibited the mitochondria-mediate apoptosis in SCI. However, since there are various apoptotic pathways such as non-caspase dependent apoptosis (Martinvalet et al., 2005), we cannot rule out the potential relationships between ICA and other apoptotic pathways.

Although the results revealed that ICA ameliorated tissue damage via inhibiting neuronal apoptosis, the mechanism might be associated with non-neuron cells. As shown in Figure 5, some NeuN^+^ cells were negative for apoptotic markers, while caspase-3 could also be positive in NeuN negative cells at the same time. Meanwhile, except for neurons, some other types of cells such as glial cells were also TUNEL positive. Therefore, it is crucial to illustrate the potential inhibitory effect of ICA on the apoptosis of non-neuronal cells. In addition, our previous study implied that prolonged ER stress induced by SCI played an important role in motor dysfunction (Wang et al., 2017b). And Li F. et al. (2015) observed that ICA inhibited ER stress-induced apoptosis of neurons through increasing the expression of synoviolin. Moreover, Wang et al., confirmed that the neuroprotection of ICA in the stroke-related brain damage was through the induction of SIRT1 by stimulating the mitogen-activated protein kinase (MAPK) pathway (Wang et al., 2009). Zhang et al. (2010) indicated that ICA suppressed oxidative stress neuropathology via the enhancement of SIRT1. Zhu et al. (2010) also suggested that ICA protected against brain ischemic injury dependent on SIRT1. Hence, it is necessary to investigate the role of ER and SIRT1 pathway in the neuroprotective effect of ICA.

In summary, the present study demonstrates that ICA especially at a high dose for early and continuous treatment may be a potent therapeutic strategy for SCI. Attenuating pro-inflammatory factors and oxidative stress via the inhibition of mitochondrial apoptotic pathway by ICA may be the underlying mechanisms of enhancing motor recovery in the mice model of SCI.

## Author Contributions

HL, KL, and LC designed the study. HL and XuZ performed the model of SCI and statistical analysis, and completed this manuscript. HL and XQ conducted the biochemical assessment. HL, XiZ, and XuZ completed the molecular biological, immunofluorescent, and histological assays. CL provided the reagent and material. KL revised the manuscript. All authors approved the manuscript.

## Conflict of Interest Statement

The authors declare that the research was conducted in the absence of any commercial or financial relationships that could be construed as a potential conflict of interest.
